# Design of a Subretinal Injection Robot Based on the RCM Mechanism

**DOI:** 10.3390/mi14111998

**Published:** 2023-10-27

**Authors:** Chenyu Yan, Manyu Liu, Guohua Shi, Jinyu Fan, Yunyao Li, Sujian Wu, Jinyuan Hu

**Affiliations:** 1 School of Biomedical Engineering (Suzhou), Division of Life Sciences and Medicine, University of Science and Technology of China, Hefei 230026, China; ycy542916022@mail.ustc.edu.cn (C.Y.);; 2 Suzhou Institute of Biomedical Engineering and Technology, Chinese Academy of Sciences, Suzhou 215163, China; 3 School of Mechanical Engineering, Beijing Institute of Technology, Beijing 100081, China

**Keywords:** RCM Institution, subretinal injection surgery, ophthalmic surgery robot

## Abstract

This study presents an investigation focusing on the advancement of a robot designed for subretinal injections in the context of macular degeneration treatment. The technique of subretinal injection surgery stands as the most efficacious approach for the successful transplantation of stem cells into the retinal pigment epithelium layer. This particular procedure holds immense significance in advancing research and implementing therapeutic strategies involving retinal stem cell transplantation. The execution of artificial subretinal surgery poses considerable challenges which can be effectively addressed through the utilization of subretinal injection surgery robots. The development process involved a comprehensive modeling phase, integrating computer-aided design (CAD) and finite element analysis (FEA) techniques. These simulations facilitated iterative enhancements of the mechanical aspects pertaining to the robotic arm. Furthermore, MATLAB was employed to simulate and visualize the robot’s workspace, and independent verification was conducted to ascertain the range of motion for each degree of freedom.

## 1. Introduction

### 1.1. Background

The successful management of cataract and corneal blindness has led to a shift in focus towards addressing retinal and optic nerve degenerative conditions as the primary challenges to human visual well-being. Notably, retinal degeneration, encompassing age-related macular degeneration (AMD) and retinitis pigmentosa (RP), has emerged as the predominant causative factor behind irreversible blindness and visual impairment on a global scale in recent years [1,2].

Retinal degenerative diseases can lead to the apoptosis of retinal cells and damage to retinal structures, which cannot be reversed. There is no effective treatment method in clinical practice. In this field of research, retinal stem cell therapy is considered one of the most promising treatment options [3]. The therapeutic efficacy of retinal stem cell transplantation has been proven by researchers from Japan [4]. However, the safety of retinal stem cell therapy cannot be guaranteed and there are few experimental samples. Therefore, further research is still needed.

Subretinal injection is an effective method to deliver the injection directly to Retinal pigment epithelium cells. It is of great significance for retinal stem cell transplantation.

There are several technical difficulties in subretinal injection surgery: ① The surgical accuracy requirement is extremely high, and far exceeds the physiological tremor amplitude of human hands. The thickness of the surgical target structure is as low as 20 µm, while the average amplitude of physiological tremors in the hands of ophthalmologists reaches 156 µm. ② Real-time positioning is difficult and requires a microscope or medical image equipment. ③ The contact force during surgery is difficult to perceive. In retinal surgery, 75% of the contact force is less than 7.5 mN. Doctors can only perceive 19% of the contact force [5,6,7].

The main problems with artificial subretinal injection surgery are as follows: ① Postoperative inflammation is more likely to occur, which can affect treatment effectiveness and even lead to surgical failure; ② it will cause more minor trauma; ③ some problems such as physiological tremors in the hands can lead to the excessive stretching of the retina and an increase in puncture hole tearing, leading to injection reflux [8,9,10].

In one study, nine surgeons used the OPMI Lumera 700 Zeiss operating microscope equipped with intraoperative optical coherence tomography to perform simulated subretinal injection surgery with and without the use of the Preceyes operating system.The result is showed in Table 1. The experiment involved using artificial retina models [10].

The sustained injection duration, needle tremor amplitude and drift distance are all key factors for successfully completing surgery. It can be seen from experimental data that the success rate and completion degree of surgery assisted by robots are significantly better than those of surgeries without robot assistance [11].

Therefore, robots are of great significance for subretinal injection surgery.

### 1.2. The Development and Current Status of Ophthalmic Surgical Robots

The remote center of motion (RCM) mechanism is of great significance for minimally invasive surgery because when the RCM point coincides with the surgical wound, the robots can move and operate within the human body without tearing the wound [12,13]. From a surgical perspective, this can improve the success rate of the surgery. In 1997, a team of researchers from Johns Hopkins University introduced the concept of utilizing remote center of motion (RCM) mechanisms in ophthalmic surgical robots [14]. Subsequently, they further advanced this concept by developing the SHER ophthalmic surgical robot. The effectiveness and potential of this robot were evaluated through retinal capsule detachment experiments conducted on an eye model. This pioneering work marked a significant milestone in the application of robotic systems for ophthalmic surgeries [9]. The Preceyes surgical robot system, developed by Eindhoven University of Technology, has emerged as a leading ophthalmic surgical robot system with global recognition. Over the course of nearly two years, starting from September 2016, a comprehensive comparative study involving six robotic surgeries and six traditional manual surgeries was conducted. Notably, in 2018, a groundbreaking milestone was achieved with the completion of the world’s first robot-assisted retinal surgery [5]. Under the skilled guidance of medical professionals, the system successfully executed retinal regeneration membrane removal surgeries on six patients, marking a significant advancement in the field of robot-assisted ophthalmic surgeries. Among the domestic research teams, the retinal surgery robot developed by Yang Yang from Beijing University of Aeronautics and Astronautics has completed vascular bypass experiments on the eyeballs of isolated animals [15,16,17]. The robot at the University of Leuven is a collaborative control platform that follows the impedance paradigm, which enables an interaction between retinal tissue and instruments [18]. Additionally, researchers from TsingHua University designed a parallel surgical robot with a remote center of motion, and the kinematic optimization design was studied. The surgical robot is composed of two branches, each of which is a planar mechanism [19].

The field of robots designed exclusively for subretinal injection surgery remains relatively underexplored, with limited research and development efforts thus far. However, researchers have made notable progress in this area by conducting simulated subretinal injection experiments utilizing the Preceyes surgical robot [7]. These experiments have demonstrated the successful validation of the surgical robot’s efficacy and enhancement in facilitating subretinal injection surgery. A group of researchers affiliated with Beijing University of Aeronautics and Astronautics conducted a series of subretinal injection surgery experiments utilizing a vascular bypass robot on pig eyeballs [11]. The aim of these experiments was to investigate the feasibility and effectiveness of the robot-assisted approach in the context of subretinal injection surgery.

Retinal stem cell therapy represents the cutting-edge and most promising treatment modality for retinal degenerative diseases. However, numerous challenges persist in the field of retinal stem cell therapy, encompassing concerns over its side effects, the induction of stem cell differentiation, and the choice of suitable vectors for cell delivery. Therefore, extensive research support is still required to address these issues comprehensively. The development of surgical robots undoubtedly holds significant implications for advancing such investigations. Presently, most experiments involving subretinal injections assisted by robots employ general-purpose surgical robots, with a limited focus on robots specifically designed for subretinal injection procedures. Moreover, in the design of robots, structural aspects are considered to be designed in conjunction with other equipment or to meet different experimental needs. Specifically, some imaging devices have shorter working distances or larger head volumes, which can easily interfere with robots. Furthermore, different experiments require punctures at different angles or positions, so we have designed a large range of motion for the robot, increased the feed distance of the end effector, and reduced the clamping angle of the end effector. In existing designs, these issues are rarely considered in the process of mechanical structure design.

### 1.3. Research Content of This Article

The focus of this study was the development of a dedicated robotic system designed specifically for subretinal injection surgery. The robot was meticulously engineered to possess three degrees of freedom that are uncoupled, meaning they can operate independently of each other. Each degree of freedom was precisely designed to meet the specific criteria and requirements of the surgical procedure.

The motion capabilities of the robot were thoroughly evaluated to ensure a satisfactory level of accuracy. This is crucial in subretinal injection surgery, where precision and exact positioning are of utmost importance. By meeting the stringent accuracy requirements, the developed robot offers a reliable and effective tool for performing subretinal injections.

The development process involved a meticulous modeling approach that combined computer-aided design (CAD) and finite element analysis (FEA). CAD allowed for the creation and visualization of a detailed digital model of the robot, enabling iterative refinements and optimizations of its mechanical aspects. FEA, on the other hand, facilitated comprehensive simulations to assess the structural integrity and performance of the robot under different conditions. This iterative process ensured that the robot’s mechanical design met the highest standards of functionality and reliability.

To further evaluate the robot’s capabilities, MATLAB was employed to simulate and visualize its workspace. This analysis provided valuable insights into the range and limitations of the robot’s movements, enabling a comprehensive understanding of its operational boundaries. Additionally, the verification of motion range was conducted separately for each individual degree of freedom, ensuring that the robot’s movements aligned precisely with the desired specifications.

The successful development of this specialized robot for subretinal injection surgery holds significant implications for the field of ophthalmology and robotic-assisted surgical procedures. By providing a reliable and accurate tool specifically designed for subretinal injections, this robot offers potential improvements in surgical precision, patient outcomes, and overall procedural efficiency. The meticulous modeling and simulation processes employed in this study contribute to the advancement of robotic-assisted surgical systems, laying the foundation for further innovations in the field.

In summary, the developed robot for subretinal injection surgery demonstrates meticulous engineering and rigorous evaluation. Its uncoupled degrees of freedom, accurate motion capabilities, and optimized mechanical design make it a valuable tool for performing precise and reliable subretinal injections. The study’s contributions to the field of ophthalmology and robotic-assisted surgical systems offer potential enhancements in surgical procedures and pave the way for future advancements in robotic surgery.

## 2. Design Scheme and Kinematics Analysis

### 2.1. Overall Design Scheme

#### 2.1.1. Design Requirements and Objectives

In the context of ophthalmic surgical procedures, it is customary for physicians to employ a cannula as their primary tool for penetrating the initial point of insertion on the sclera. Subsequently, the instrument is introduced into the eye by traversing along the path defined by the cannula. Minimizing the size of the scleral opening is of paramount importance due to the associated advantages of expedited wound healing and reduced susceptibility to infection. In certain cases, when the incision is sufficiently small, the application of sutures becomes unnecessary. To attain a diminutive aperture, it is imperative to minimize the forces exerted upon the scleral opening. Consequently, the surgical instrument should be guided with the scleral opening serving as the focal point of motion, ensuring that all axes of movement converge at this pivotal juncture. For the RCM mechanism used in this article, iris incision is the RCM point.

**Figure 1:**
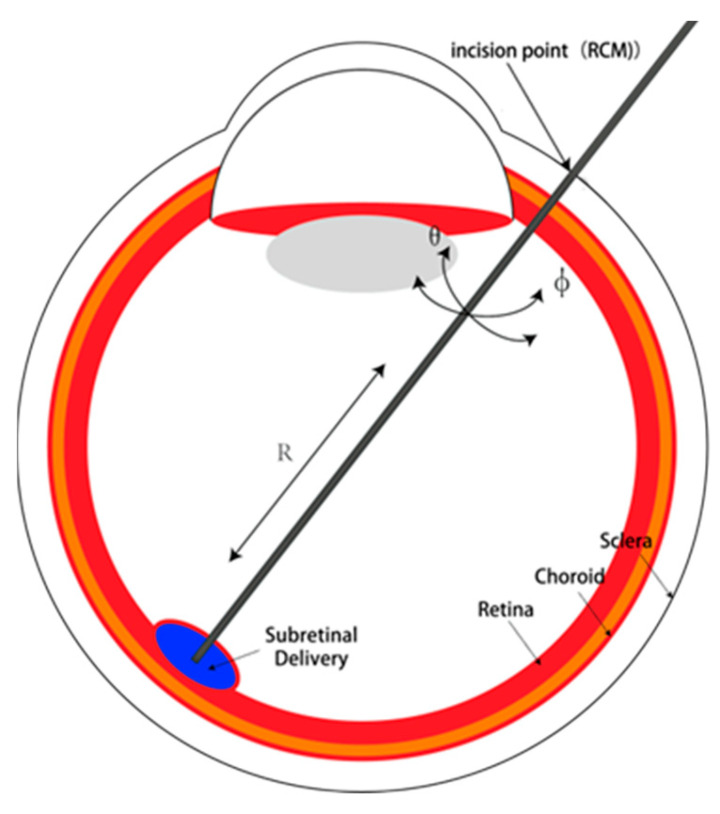
Degrees of freedom of surgical instruments.

The primary design imperative for an ophthalmic surgical robot lies in guaranteeing its motion capabilities during surgical procedures. This necessitates the incorporation of six essential degrees of freedom. Specifically, three translational degrees of freedom are indispensable for the RCM (remote center of motion) point of the mechanism, enabling the precise alignment of the RCM point with the iris incision. The remaining three degrees of freedom are pivotal in ensuring the robot’s mobility within the eye. These encompass the axial feed degree of freedom for the surgical actuator, as well as the lateral and longitudinal rotations around the RCM point. The Degrees of freedom of surgical instruments is showed in Figure 1.

Motion accuracy stands as a paramount design criterion in ophthalmic surgery, particularly given the minute scale of the surgical target structures, reaching as low as 20 µm. As a result, ensuring adequate precision becomes imperative. This article, however, does not delve into the construction and investigation of control systems. Instead, the emphasis lies in guaranteeing satisfactory motion accuracy through meticulous mechanical design strategies. Notably, special attention will be paid to enhancing the precision of single-step motions. Techniques such as motion scaling will be employed to amplify the precision of the output motion relative to its input, and using the screw drive to improve transmission accuracy and reliability.

The workspace of a robot is another crucial factor that requires consideration in robot design. The workspace refers to the overall volume traversed by the end effector when the robot performs all possible motions. The workspace is constrained by the robot’s geometric structure and mechanical limits on its joints, serving as a critical indicator for evaluating the performance of the robotic arm. There are three main methods for analyzing the robot’s workspace: geometric construction, analytical methods, and numerical computation. In this study, the robot’s motion is determined by three independent degrees of freedom, making the workspace analysis problem relatively straightforward. Consequently, a combination of analytical methods and geometric construction will be utilized to solve the workspace-related problems, followed by simulation and visualization using Matlab2023a software.

#### 2.1.2. Design Scheme

The foundational element in the robotic system serves as a crucial link between the rear robotic arm and the rest of the system. It allows for the adjustment of the remote center of motion (RCM) point position, enabling precise alignment with the iris incision. This adjustment is essential for achieving accurate and controlled movements during surgical procedures.

RCM mechanisms are of great significance for minimally invasive surgery because when the RCM point coincides with the surgical wound, robots can move and operate within the human body without tearing the wound. From a surgical perspective, this can improve the success rate of the surgery. Unlike robots that use RCM mechanisms, those that do not use RCM mechanisms need algorithms to control their motion to avoid tearing wounds. This is not only more difficult, but also more dangerous.

The base turntable is responsible for facilitating rotational degrees of freedom within the XZ plane. It provides the necessary rotational motion for the robot, allowing it to maneuver and position itself effectively during the surgical procedure.

The RCM mechanism, on the other hand, enables pitch degrees of freedom within the YZ plane. This mechanism allows the robot to perform controlled pitch movements, which are valuable for accessing and maneuvering around the surgical target area.

The end effector is the component responsible for executing specific tasks such as end feed and injection. It is equipped with an actuator that drives the necessary movements for these tasks. The actuator plays a vital role in ensuring the precise and controlled motion of the end effector, enabling the accurate positioning and manipulation of surgical tools.

To enhance motion accuracy, both the RCM mechanism and the end effector utilize screw drives as their driving mechanism. Screw drives offer several advantages, including improved transmission accuracy and reliability, which are crucial for achieving precise and repeatable motions in surgical procedures.

By incorporating screw drives into the RCM mechanism and end effector, the robotic system can enhance its overall motion accuracy, ensuring that surgical tasks are performed with precision and minimal errors. These design choices contribute to the system’s effectiveness and reliability in ophthalmic surgery.

### 2.2. Kinematic Analysis

**Figure 2:**
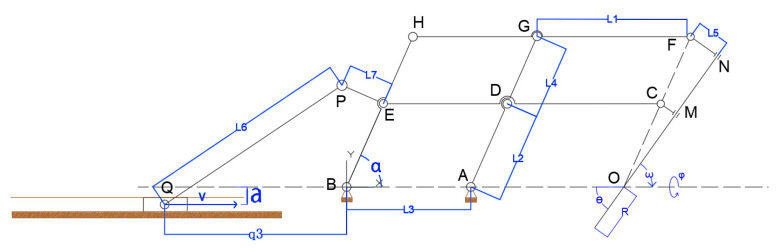
Mechanism diagram—YZ plane.

**Figure 3:**
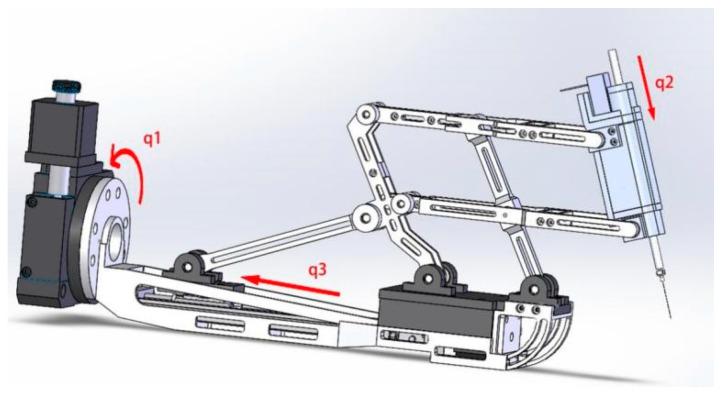
Mechanical structure.

In terms of configuration design, a dual parallel quadrilateral configuration is adopted to ensure the stability of the system. The robot’s motion around the X axis is achieved through a dual parallel quadrilateral mechanism. On the reference plane, YZ, the translation of Q is driven by a screw mechanism, which in turn actuates the link HEPB through the driving rod PQ, resulting in the motion of the quadrilateral BAGH. The variation in angle ABH is reflected in the quadrilateral HECF, thereby driving the rotation of CF. The axis of the end effector forms a constraining angle with CF and passes through the designated remote center of motion (RCM) point O. On the other hand, the robot’s motion around the Y axis is directly driven by a rotary motor, while the needle axis of the end effector is driven by a screw mechanism.The Mechanism digram of the robot is showed in Figure 2 and the mechanical structure is showed in Figure 3.

The length of each rod is $L_{1}$–$L_{7}$. The distance between the lead screw and the RCM mechanism base is a. Angle HBA is the angle of α. The turning angle of the turntable is $q_{1}$, the axial feed distance of the syringe is $q_{2}$, and the movement of the lead screw slider in the RCM mechanism is $q_{3}$.The three degrees of freedom of the end effector are the lateral swing angle, φ, pitch, θ, end axial feed R, and then

(1) $$\varphi =q_{1}$$

(2) $$R=q_{2}$$

(3) $$\theta =\alpha -\sin^{-1}\frac{l_{5}}{l_{2}+l_{4}}$$

#### 2.2.1. Forward Kinematics

Let

(4) $$\beta =\alpha +\tan^{-1}\frac{l_{7}}{l_{2}}=\theta +\sin^{-1}\frac{l_{5}}{l_{2}+l_{4}}+\tan^{-1}\frac{l_{7}}{l_{2}}$$

Based on geometric relationships, it is possible to deduce or infer a certain conclusion:

(5) $${\left(\mathrm{PB}+\frac{a}{\sin\beta }\right)}^{2}+{\left(q-\frac{a}{\tan\beta }\right)}^{2}+2*(\mathrm{PB}+\frac{a}{\sin\beta })(q-\frac{a}{\tan\beta })\cos\beta =l_{6}{}^{2}$$

According to Equations (1)–(5),

(6) $$\theta =\pi -\sin^{-1}\left(\frac{l_{6}{}^{2}-\left(l_{2}{}^{2}+l_{7}{}^{2}\right)-a^{2}-q_{3}{}^{2}}{2\times \sqrt{a^{2}+q_{3}{}^{2}}\times \sqrt{l_{2}{}^{2}+l_{7}{}^{2}}}\right)-\tan^{-1}\frac{q_{3}}{a}-\sin^{-1}\frac{l_{5}}{l_{2}+l_{4}}-\tan^{-1}\frac{l_{7}}{l_{2}}$$

#### 2.2.2. Inverse Kinematics

Based on geometric relationships and Equations (1)–(5), it can be concluded that

(7) $$\beta =\pi -\sin^{-1}\left(\frac{l_{6}{}^{2}-\left(l_{2}{}^{2}+l_{7}{}^{2}\right)-a^{2}-q_{3}{}^{2}}{2\times \sqrt{a^{2}+q_{3}{}^{2}}\times \sqrt{l_{2}{}^{2}+l_{7}{}^{2}}}\right)-\tan^{-1}\frac{q_{3}}{a}$$

The following is obtained after substitution:

(8) $$\begin{array}{ll} q_{3} & =\sqrt{\begin{array}{c} {l_{6}}^{2}-\left({l_{2}}^{2}+{l_{7}}^{2}\right)-a^{2}+\left({l_{2}}^{2}+{l_{7}}^{2}\right)\cos{\left(\theta -\sin^{-1}\frac{l_{5}}{l_{2}+l_{4}}+\tan^{-1}\frac{l_{7}}{l_{2}}\right)}^{2}\\ -2a\times \sqrt{{l_{2}}^{2}+{l_{7}}^{2}}\times \sin\left(\theta -\sin^{-1}\frac{l_{5}}{l_{2}+l_{4}}+\tan^{-1}\frac{l_{7}}{l_{2}}\right) \end{array}}\\ & -\sqrt{{l_{2}}^{2}+{l_{7}}^{2}}\times \cos(\theta -\sin^{-1}\frac{l_{5}}{l_{2}+l_{4}}+\tan^{-1}\frac{l_{7}}{l_{2}}) \end{array}$$

#### 2.2.3. Differential Kinematics

The pitch motion speed at the end of the actuator is ω, and the motion speed of point Q is v. According to Equation (6),

(9) $$\omega =\frac{v}{\sqrt{l_{2}{}^{2}+l_{7}{}^{2}}}\times \left(l_{6}{}^{2}+{\left(q_{3}-\frac{a}{A}\right)}^{2}-{\left(\sqrt{l_{2}{}^{2}+l_{7}{}^{2}}+\frac{a}{B}\right)}^{2}\right)÷\left(2\times {\left(q_{3}-\frac{a}{A}\right)}^{2}\times B\right)$$

In Equation (9),

$$A=\tan(\theta +\sin^{-1}\frac{l_{5}}{l_{2}+l_{4}}+\tan^{-1}\frac{l_{7}}{l_{2}})\;\;\;B=\sin(\theta +\sin^{-1}\frac{l_{5}}{l_{2}+l_{4}}+\tan^{-1}\frac{l_{7}}{l_{2}})$$

### 2.3. Mechanical Design and Selection

By taking into account factors such as facial size, eyeball size, load requirements, and external dimensions of the human body, the dimensions are established through a comprehensive determination process [9]. The size of Robot size paramaeters are showed in Table 2.

**Figure 4:**
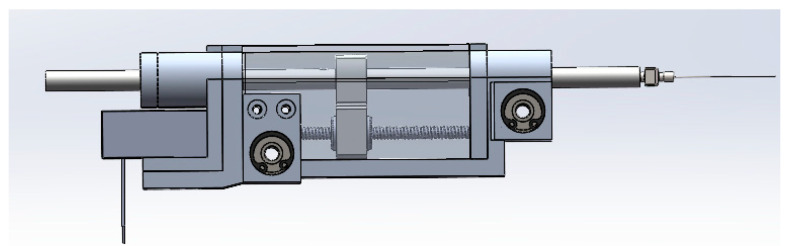
End effector.

The mechanical structure is shown in the Figure 4.

In the RCM (remote center of motion) structure, the control of the robot’s motion relies on three input variables, namely q_1_, q_2_, and q_3_, which are associated with three distinct motors. These motors play crucial roles in enabling specific types of movements for the robot.

The first motor, integrated with the rotary table and the gear–rack mechanism, directly drives the robot’s rotational motion around the RCM point. This input variable, denoted as q_1_, ensures precise control over the rotation of the robot.

The second motor, located within the end effector and coupled with a screw structure, governs linear motion, denoted as q_2_. This linear motion is responsible for controlling the feed motion of the end effector. By accurately manipulating q_2_, the robot can achieve precise and controlled linear movements.

The third motor, positioned beneath the RCM mechanism, is combined with a screw mechanism to generate the linear motion q_3_. This specific linear motion is instrumental in controlling the pitch motion of the robot around the RCM point. By skillfully manipulating q_3_, the robot can execute controlled pitch movements.

To assess the motion accuracy of each degree of freedom, computational methods are employed. By analyzing the drive modes associated with each motor, it becomes possible to calculate and determine the respective precision of each degree of freedom. This evaluation allows for a comprehensive understanding of the robot’s overall motion performance and facilitates the optimization of its motion control system.

Three motors drive q_1_, q_2_, and q_3_ to control the three end degrees of freedom of the robot. Given the workspace considerations, particularly the limited working distance of commonly employed optical coherence tomography equipment in subretinal surgery, a broader range of degrees of freedom is necessary to accommodate multiple postures during operation. Consequently, the values assigned to each degree of freedom for the robotic system are showed in Table 3.

## 3. Mechanical Analysis and Simulation

### 3.1. Mechanical Analysis of Robots

**Figure 5:**
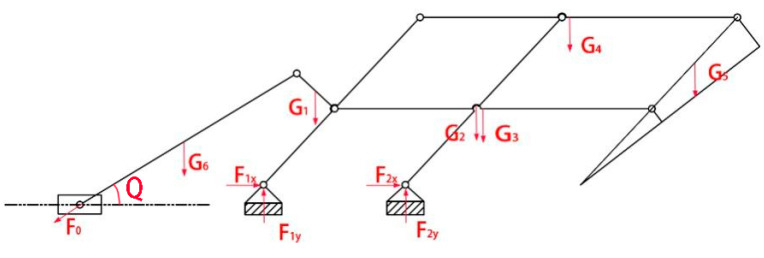
Mechanical force analysis.

To facilitate mechanical simulation, an initial step involves conducting a comprehensive mechanical analysis of the two distinct working postures assumed by the robotic system. This analysis aims to generate essential data that will contribute to the subsequent simulation process. The Mechanical force analysis is showed in Figure 5. 

The force system equation for this posture is as follows:

(10) $$F_{0}\times \cos Q=F_{1X}+F_{2X}$$

(11) $$G_{0}=F_{1Y}+F_{2Y}+F_{0}\times \sin Q$$

(12) $$F_{1Y}q_{3}+F_{2Y}\times \left(q_{3}+l_{3}\right)+(F_{1X}+F_{2X})a=G_{1}(\frac{l_{7}}{2}\sin\alpha +l_{6}\cos Q)+\;G_{2}\left(l_{3}-\frac{l_{2}+l_{4}}{2}\cos\alpha +q_{3}\right)+G_{3}\left(\frac{l_{1}+l_{3}}{2}-l_{2}\cos\alpha +q_{3}\right)+\;G_{4}\left[\frac{l_{1}+l_{3}}{2}-\left(l_{2}+l_{4}\right)\cos\alpha +q_{3}\right]+G_{5}\left[l_{2}+l_{4}-(l_{2}+\frac{l_{4}}{2})\cos\alpha +q_{3}\right]G_{6}\frac{l_{6}\cos Q}{2}$$

(13) $$G_{1}\left(l_{2}\cos\alpha +\frac{l_{7}}{2}\sin\alpha \right)+G_{6}\left(q_{3}-\frac{l_{6}\cos Q}{2}\right)+F_{2Y}l_{3}\;\;=F_{0}\left[\left(q_{3}+\frac{a}{\tan Q}\right)\sin Q-\frac{a}{\cos Q}\right]+G_{2}\left(l_{3}-\frac{l_{2}+l_{4}}{2}\cos\alpha \right)+G_{3}\left(\frac{l_{1}+l_{3}}{2}-\frac{l_{2}+l_{4}}{2}\cos\alpha \right)+\;G_{4}\left[\frac{l_{1}+l_{3}}{2}-\left(l_{2}+l_{4}\right)\cos\alpha \right]+G_{5}\left[l_{2}+l_{4}-\left(l_{2}+\frac{l_{4}}{2}\right)\cos\alpha \right]$$

### 3.2. Mechanical Simulation

Based on the outcomes of the mechanical analysis, a subsequent step involves conducting finite element analysis on the robotic system in the pose state where the highest forces are experienced. This analysis aims to provide a detailed understanding of the structural behavior and stress distribution within the robot, offering valuable insights into its mechanical performance under demanding conditions.

#### Mechanics Simulation

**Figure 6:**
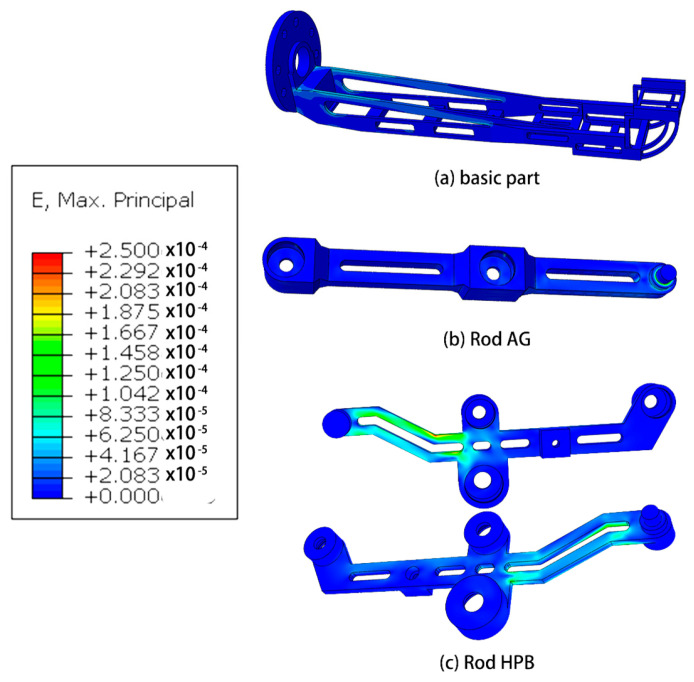
Simulation of strain of some parts.

**Figure 7:**
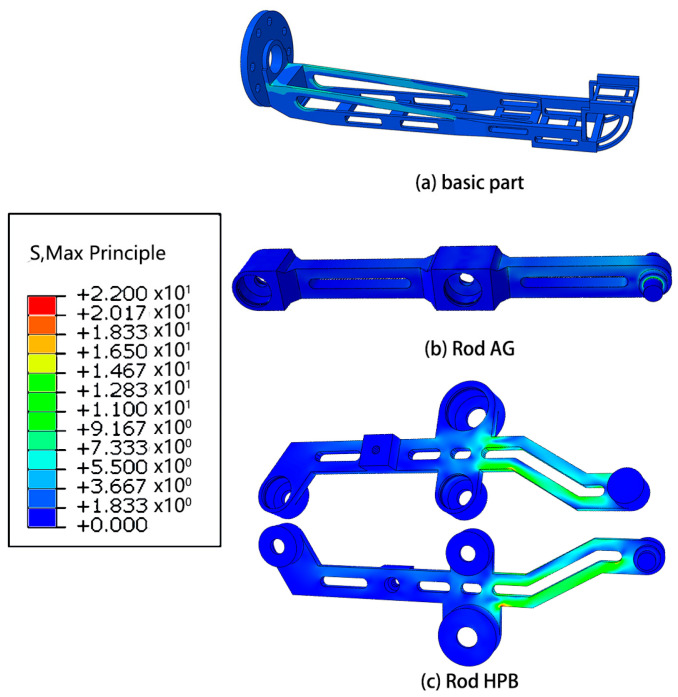
Simulation of stress of some parts.

**Figure 8:**
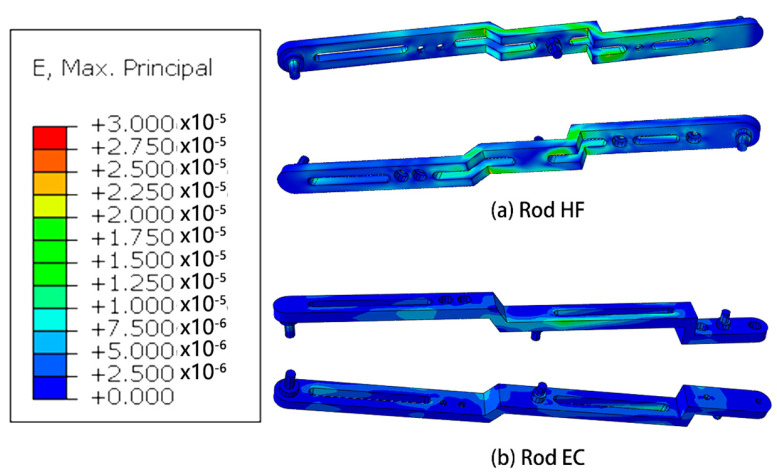
Simulation of strain of some rods.

**Figure 9:**
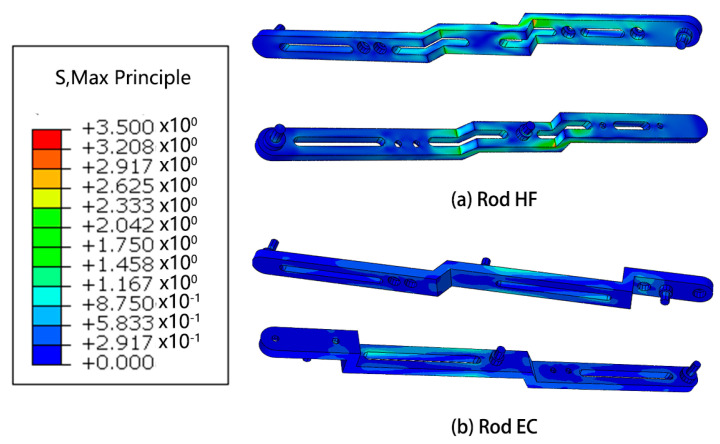
Simulation of stress of some rods.

Results of finite element analysis of mechanical structures are showed in Figure 6, Figure 7, Figure 8 and Figure 9.

The research conducted in this study involved the utilization of finite element analysis to evaluate the mechanical performance of the designed robot. The results obtained from this analysis indicate that the robot demonstrates favorable mechanical characteristics. This suggests that the robot is capable of effectively fulfilling its intended functions and requirements.

Furthermore, the analysis reveals that there is considerable scope for optimization in the design of the robot. This implies that there are opportunities to further enhance the robot’s mechanical performance, potentially leading to improvements in areas such as structural integrity, load-bearing capacity, or overall efficiency. These optimization possibilities present avenues for future research and development efforts.

## 4. Verification of Robot Working Posture

### 4.1. Robot Motion Accuracy

The investigation of control accuracy is beyond the scope of the present study and will be explored in forthcoming articles. The current research is solely focused on the verification of the robot’s motion accuracy, excluding any analysis or evaluation of its control performance.

Table 4 presented herein showcases the motion accuracy of robots, which was derived from a careful consideration of the parameters and mechanical structure pertaining to each motor. The provided data offer insights into the precise motion capabilities of the robots under investigation.

### 4.2. Workplace of the Robot

**Figure 10:**
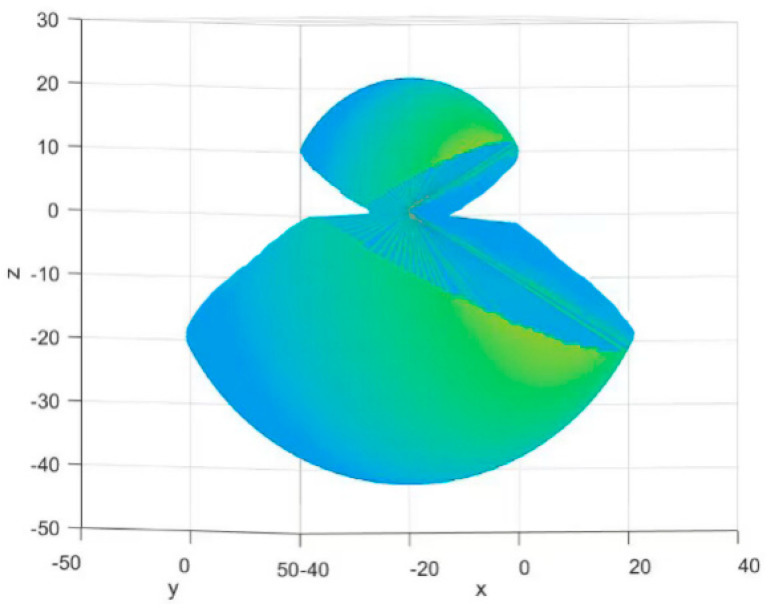
Visualization of workspace of the robot.

We used MATLAB for work simulation and constructed a workspace visualization diagram. The three translational DoFs of the end effector [x, y, z], T, are taken as the description coordinates of the reachable workspace. It’s showed in Figure 10.

**Figure 11:**
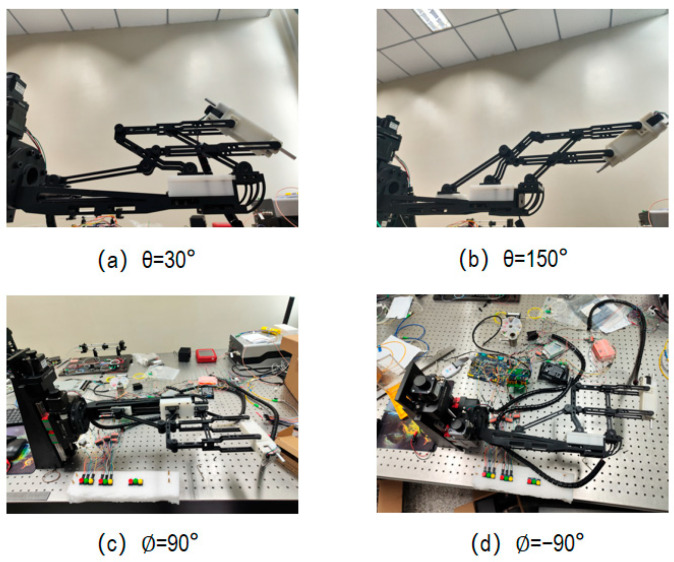
The boundary of workspace of the robot.

The motion of the needle tip is determined via three uncoupled degrees of freedom, so we just verified the range of motion on each degree of freedom separately (Figure 11).

## 5. Discussion

Retinal stem cell therapy represents the cutting-edge and most promising treatment modality for retinal degenerative diseases. However, numerous challenges persist in the field of retinal stem cell therapy, encompassing concerns over its side effects, the induction of stem cell differentiation, and the choice of suitable vectors for cell delivery. Therefore, extensive research support is still required to address these issues comprehensively. The development of surgical robots undoubtedly holds significant implications for advancing such investigations. Presently, most experiments involving subretinal injections assisted by robots employ general-purpose surgical robots, with a limited focus on robots specifically designed for subretinal injection procedures. Moreover, existing subretinal injection surgical robots seldom consider integration with auxiliary equipment such as optical devices and lack comprehensive support for various experimental requirements. The robot presented in this study demonstrates excellent support for subretinal injection procedures.

The primary focus of this study is the development of a surgical robot that offers an expanded workspace. The aim is to accommodate diverse experimental conditions, particularly in the context of subretinal injections. Currently, there is a scarcity of research specifically dedicated to investigating the workspace requirements for subretinal injections. Additionally, there is no established consensus regarding the recommended surgical area within the fundus.

To address these gaps in knowledge, we intend to leverage the developed surgical robot and collaborate with our well-established optical imaging system. By combining the capabilities of the robot and the imaging system, we plan to conduct a series of experiments. These experiments will primarily focus on determining the optimal surgical area for subretinal injection procedures, with a specific emphasis on identifying the puncture zone within the fundus. The outcomes of these experimental investigations will provide valuable insights into the appropriate surgical region for subretinal injections. Based on the data obtained from these experiments, we will then proceed to make specialized design modifications to the robot’s working area. These modifications will be tailored to enhance the robot’s performance and ensure optimal functionality within the identified surgical area. Ultimately, the integration of the surgical robot, optical imaging system, and the findings from the experimental investigations will contribute to advancing the field of subretinal injections. The research outcomes will inform and guide future surgical procedures, facilitating improved precision and success rates in subretinal injection procedures.

Furthermore, the exploration of other pertinent aspects of subretinal injection surgery warrants significant research attention. For instance, investigating the impact of different puncture angles on the outcomes of the procedure holds considerable relevance. The findings from these studies will also guide the iterative development of surgical robots.

Our forthcoming research endeavors will be delineated into two key domains. Firstly, from a mechanical perspective, we believe that more consideration should be given to the practical problems encountered during surgery instead of designing new institutions. For example, we can use a mechanical structure to connect the endoscopic OCT device with the end effector of the robot, or design a small mechanism on the end effector to reduce injection backflow during surgery. It also worth considering adopting replaceable end effectors. Secondly, from a control perspective, there are also many issues worth studying. There is a problem with remotely controlled robots during the surgical process: the coordinate system of the image obtained through the imaging system does not coincide with the spatial coordinate system of the operator. Therefore, how to identify and convert coordinates is a major issue. In addition, there is an angle between the needle head and the needle tube used for subretinal injection, so it is crucial to strictly control the movement of the needle head during the injection process through algorithms and extend the axial feed of the needle head.

## 6. Conclusions

Within this research article, a robot specifically tailored for subretinal injection surgery was developed. The designed robot encompasses three degrees of freedom that are uncoupled, with each degree of freedom satisfying the designated design criteria. The robot’s motion capabilities exhibit a satisfactory level of accuracy, adequately meeting the requirements of subretinal injection surgery. The modeling process involved a meticulous simulation phase utilizing both computer-aided design (CAD) and finite element analysis (FEA). This enabled the iterative refinement of the mechanical aspects of the robotic arm. Moreover, MATLAB was employed to simulate and visualize the robot’s workspace, while the verification of motion range was conducted separately for each individual degree of freedom. In the following research article, we will establish a detailed control system for the robot and then calibrate it to ensure optimal control accuracy.

We use RCM with dual-parallelogram mechanisms, which can provide higher stability and motion accuracy for the robot. Our robot moves directly through remote control. Compared to works that also use parallelogram RCM mechanisms, we add a crank mechanism to drive the motion of the parallelogram mechanism, providing a larger range of motion and higher driving accuracy. Overall, our robots have a larger range of motion, higher motion accuracy, and better structural stability compared to those in similar work. In conclusion, our robot can perform different surgeries and experiments under more conditions, so we believe that the significance of our work lies in the ability to experiment with and validate the questions of subretinal injection and retinal stem cell therapy.

## Figures and Tables

**Table 1:** Experimental results of simulated subretinal injection.

	Robot Group	Labor Group
Successful surgery (case)	8	4
Tearing of puncture hole (case)	0	7
Average injection duration (second)	52	29
Needle tip tremor amplitude (µm)	1–11, Median 1	4–266, Median 18
Needle drift distance (µm)	4–58, Median 16	115–335, Median 212

**Table 2:** Robot size parameters.

Size	Parameter Value
l_1_	105
l_2_	62.5
l_3_	85
l_4_	50
l_5_	22.5
l_6_	152
l_7_	31

**Table 3:** Range of degrees of freedom.

Degrees of Freedom	Range of Motion
q_1_	−90°~+90°
q_2_	60 mm
q_3_	125 mm
φ	−90°~+90°
R	60 mm
θ	30°~150°

**Table 4:** The motion accuracy of robots.

Input	Precision	Repeatability	Maximum Needle Tip Movement
q_1_	0.0002°	±0.005°	0.00015 mm
q_2_	0.005 mm	±0.01 mm	0.005 mm
q_3_	0.0025 mm	±0.007 mm	0.00018 mm

## Data Availability

There are no data to be shared.
